# Supplementary material for: Complexome profiling of the Chlamydomonas psb28 mutant reveals TEF5 as an early PSII assembly factor
Source: Plant Cell. 2025 Mar 17;37(6):koaf055. doi: 10.1093/plcell/koaf055 (PMC12164590; doi:10.1093/plcell/koaf055)
Supplement: koaf055_Supplementary_Data [file koaf055_supplementary_data.zip › psb28_interactive.zip!/plots/intensity/qualityPlots/peptides/TATC_Cre03.g145887.t1.1.html]

### Cre03.g145887.t1.1

Trivial name: TATC  
SeqCoverage [median]: 7.00  
Mass: 38.58 kDa  
  
 

| Sequence | MissedCleavage | Mass [Da] | wtIntensity | muIntensity | Score |
| --- | --- | --- | --- | --- | --- |
| EEVPGAQSIMR | 0 | 1215.59 | 0.00 | 355.56 | 84.21 |
| LIEQGR | 0 | 714.40 | 0.00 | 1226.90 | 90.94 |
| LIEQGRGQTVANSA | 1 | 1442.75 | 861.75 | 840.91 | 135.60 |
|  |  |  | 861.7 | 2423.4 |  |
